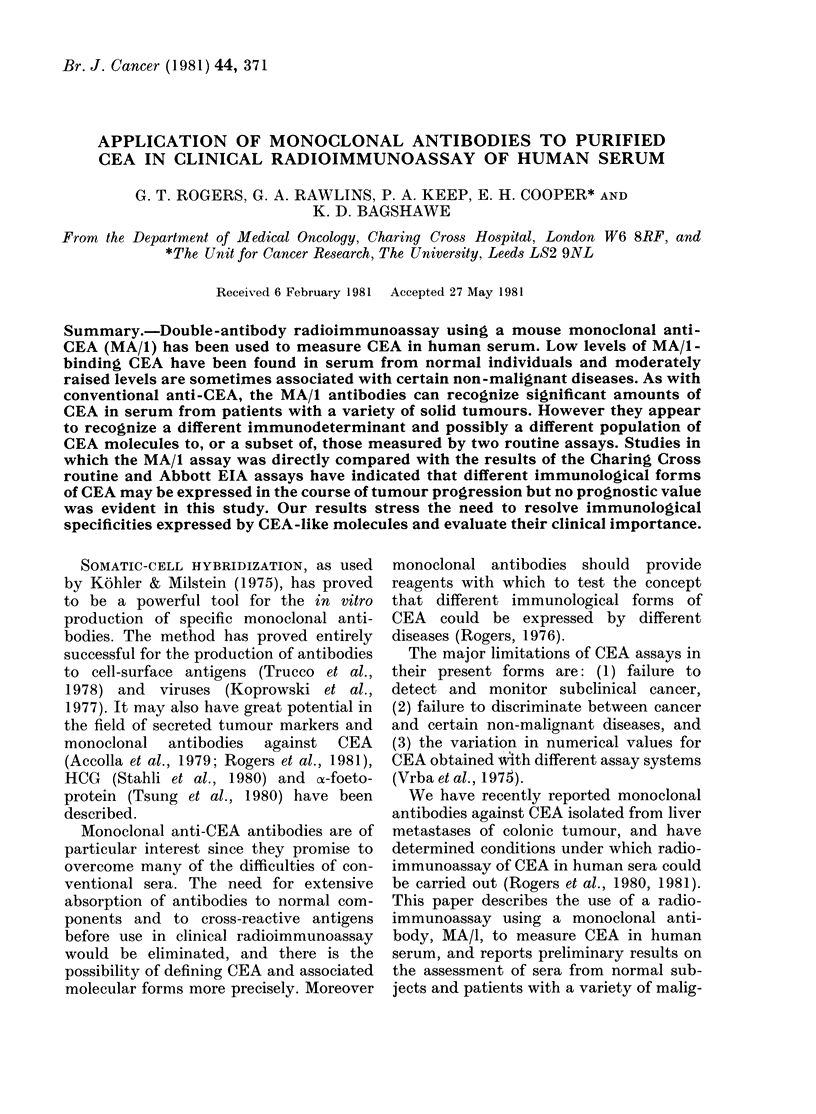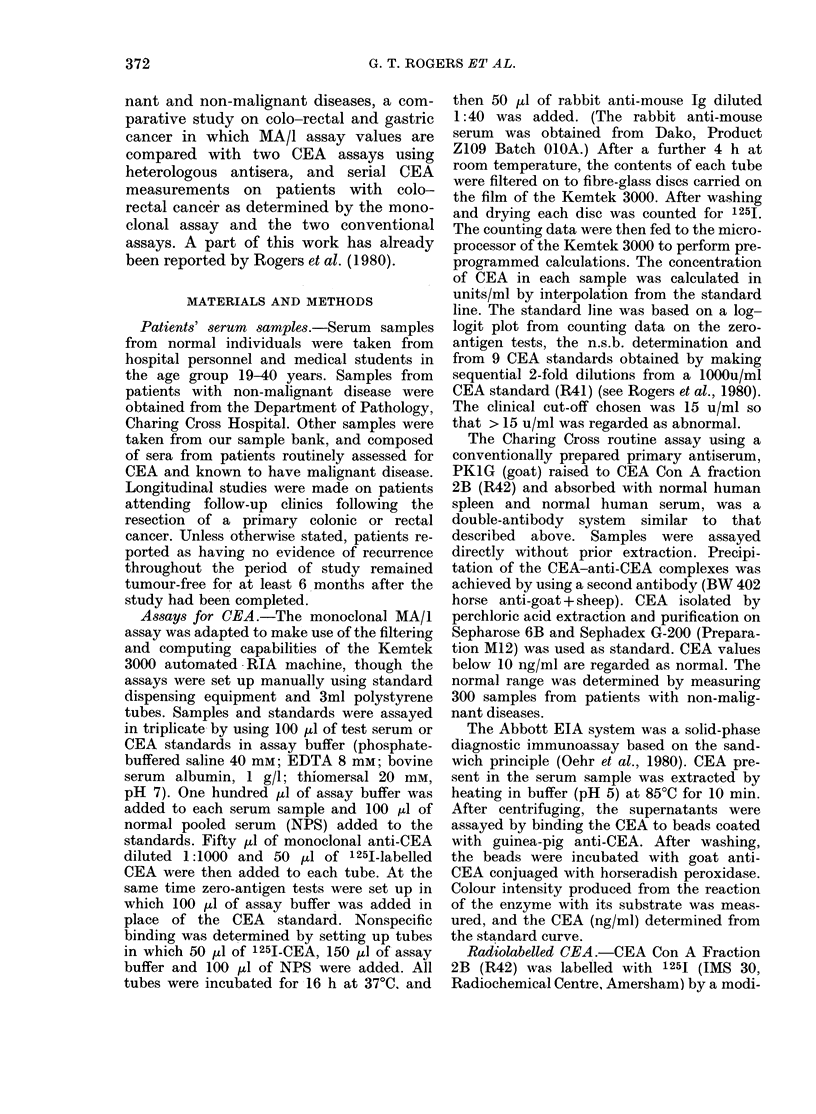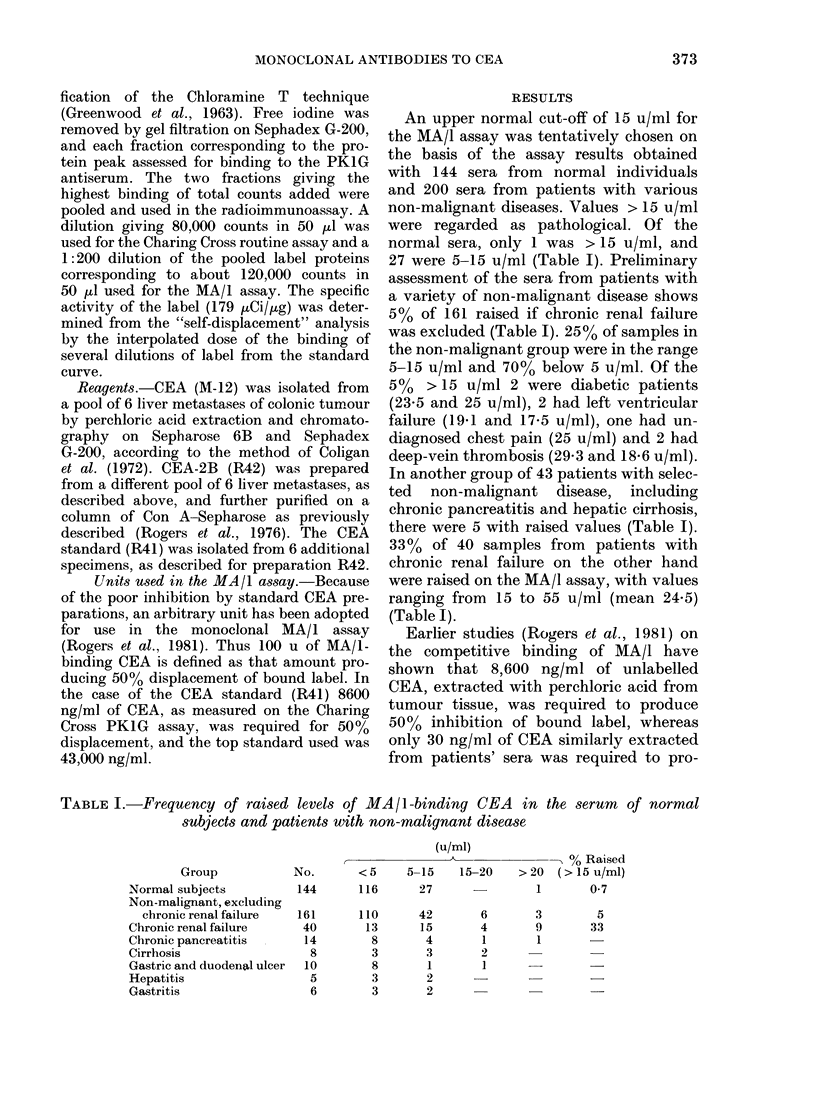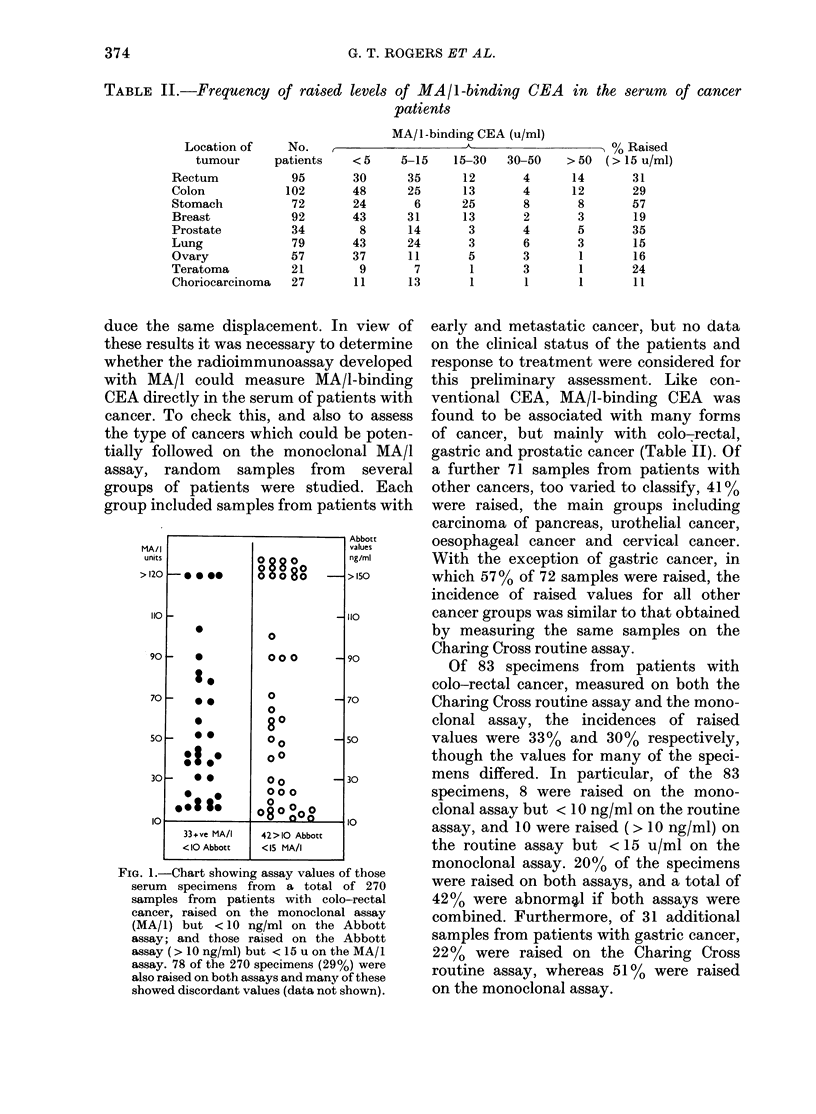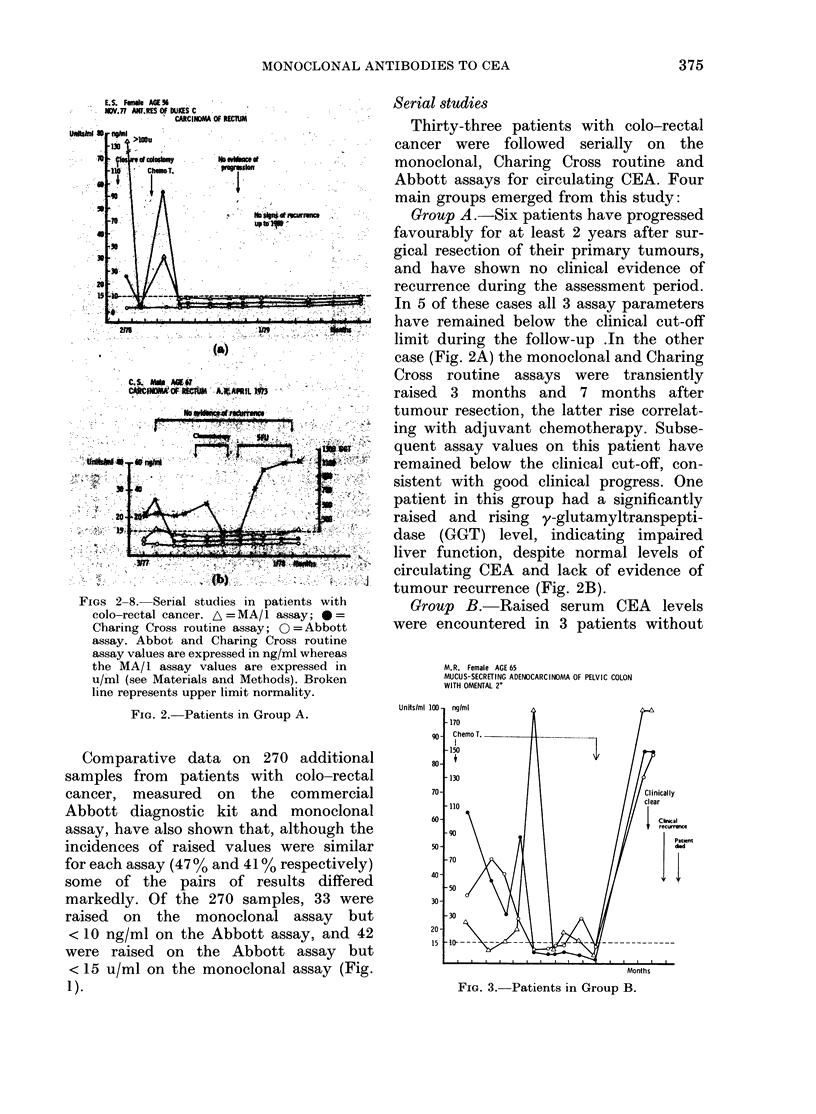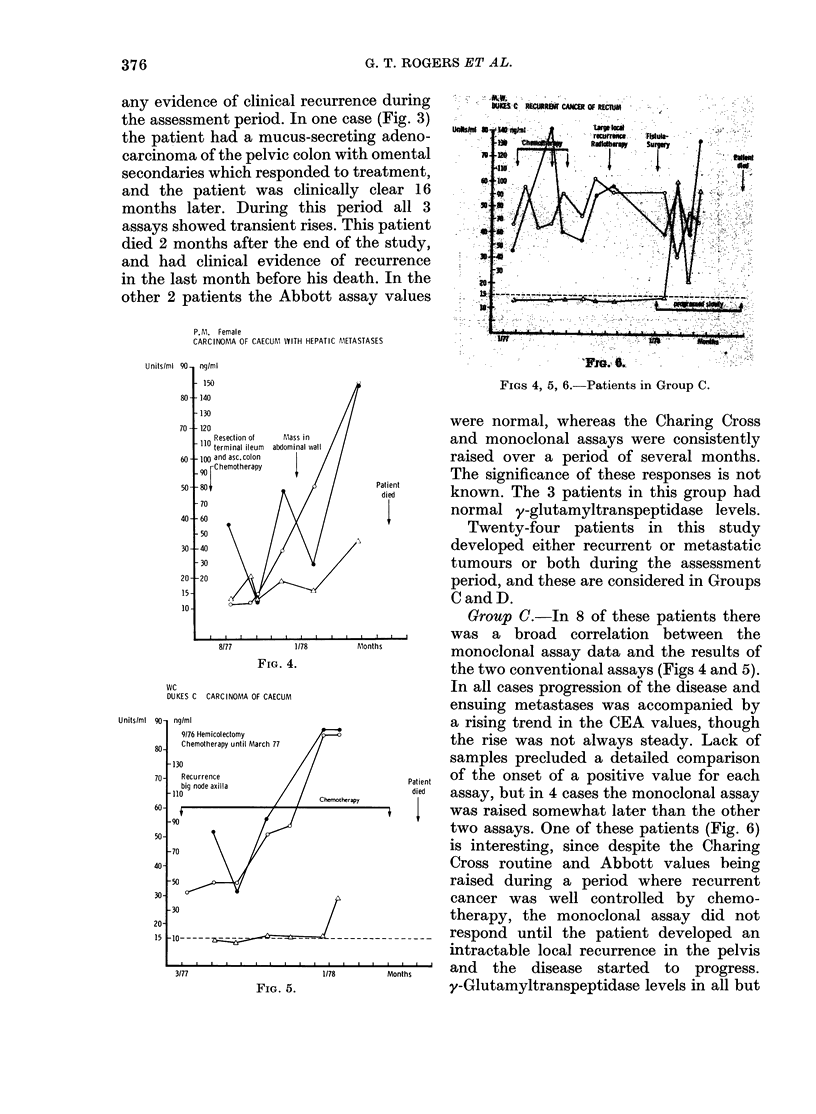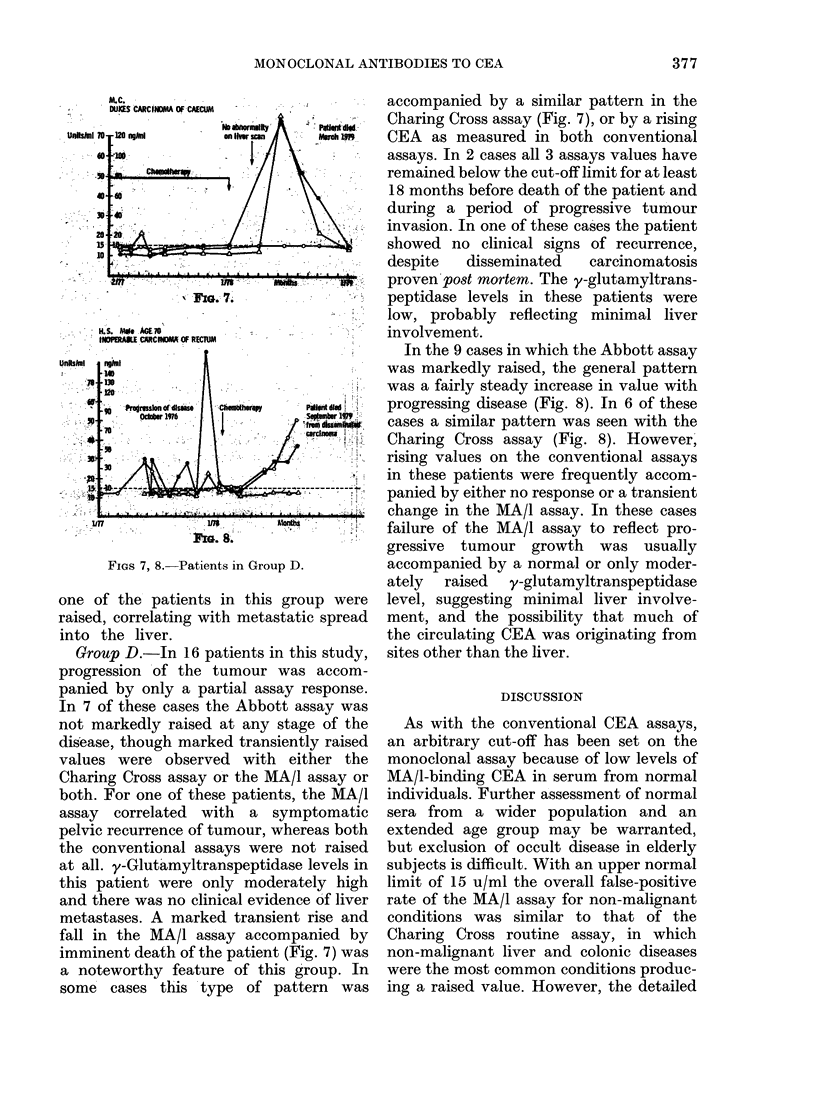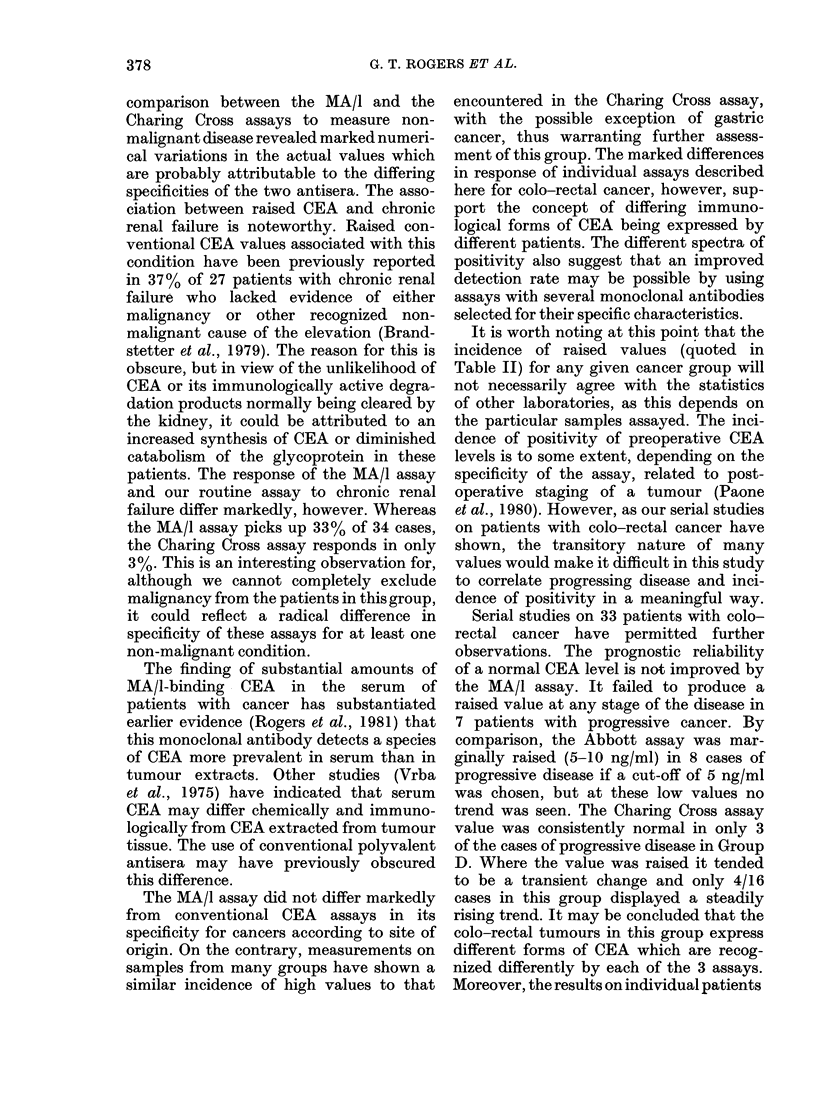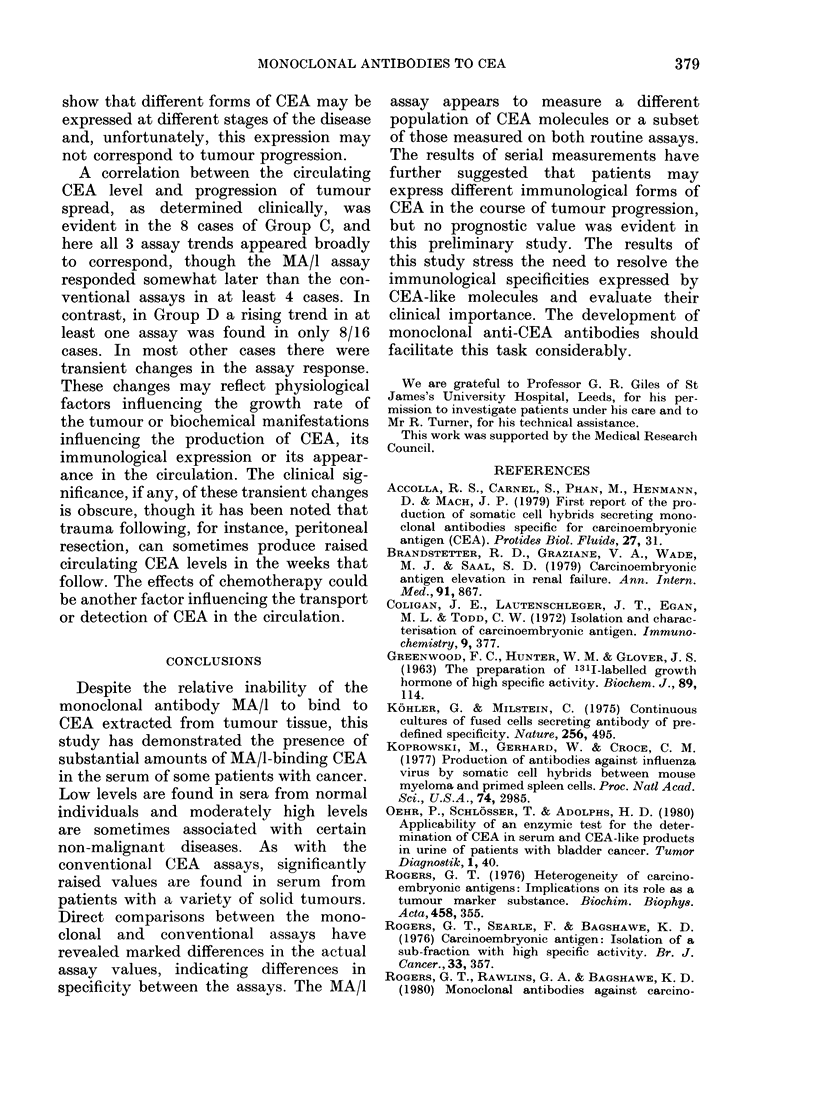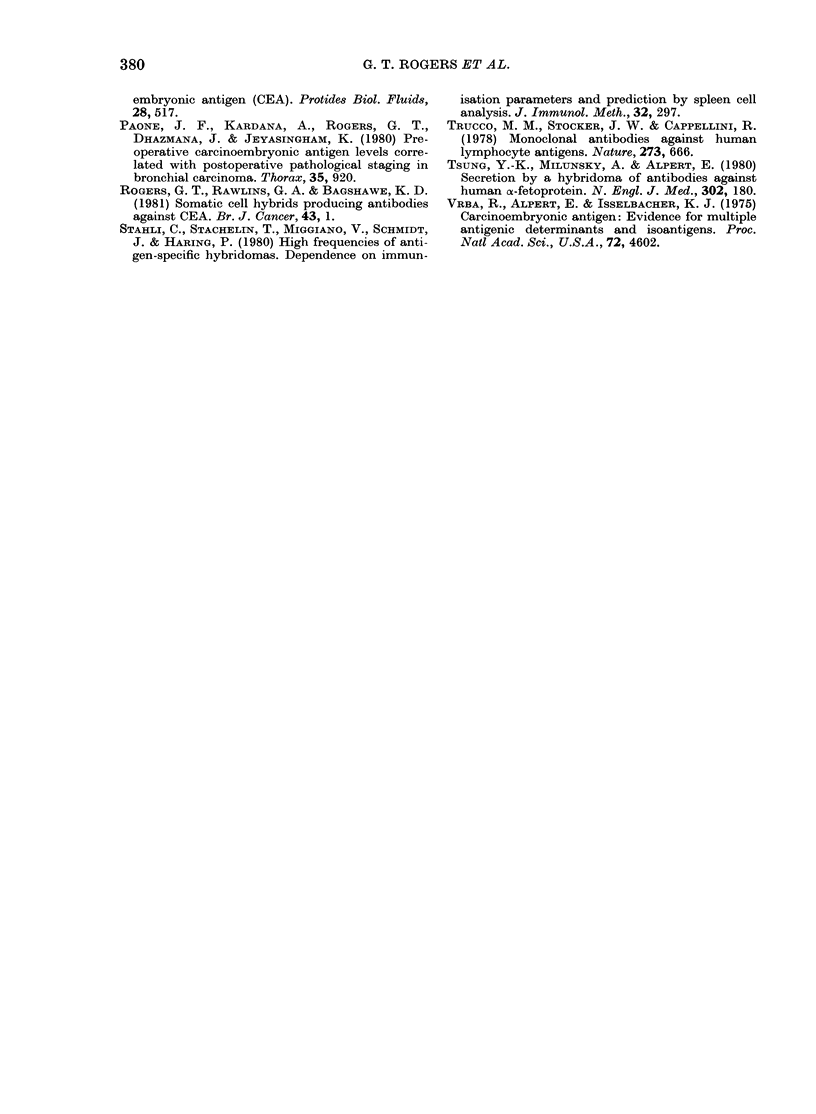# Application of monoclonal antibodies to purified CEA in clinical radioimmunoassay of human serum.

**DOI:** 10.1038/bjc.1981.194

**Published:** 1981-09

**Authors:** G. T. Rogers, G. A. Rawlins, P. A. Keep, E. H. Cooper, K. D. Bagshawe

## Abstract

Double-antibody radioimmunoassay using a mouse monoclonal anti-CEA (MA/1) has been used to measure CEA in human serum. Low levels of MA/1-binding CEA have been found in serum from normal individuals and moderately raised levels are sometimes associated with certain non-malignant diseases. As with conventional anti-CEA, the MA/1 antibodies can recognize significant amounts of CEA in serum from patients with a variety of solid tumours. However they appear to recognize a different immunodeterminant and possibly a different population of CEA molecules to, or a subset of, those measured by two routine assays. Studies in which the MA/1 assay was directly compared with the results of the Charing Cross routine and Abbott EIA assays have indicated that different immunological forms of CEA may be expressed in the course of tumour progression but no prognostic value was evident in this study. Our results stress the need to resolve immunological specificities expressed by CEA-like molecules and evaluate their clinical importance.


*Br. J. Cancer* (1981) **44**, 371

### APPLICATION OF MONOCLONAL ANTIBODIES TO PURIFIED CEA IN CLINICAL RADIOIMMUNOASSAY OF HUMAN SERUM

G. T. ROGERS, G. A. RAWLINS, P. A. KEEP, E. H. COOPER* AND K. D. BAGSHAWE

*From the Department of Medical Oncology, Charing Cross Hospital, London W6 8RF, and *The Unit for Cancer Research, The University, Leeds LS2 9NL*

Received 6 February 1981 Accepted 27 May 1981

**Summary.—Double-antibody radioimmunoassay using a mouse monoclonal anti-CEA (MA/1) has been used to measure CEA in human serum. Low levels of MA/1-binding CEA have been found in serum from normal individuals and moderately raised levels are sometimes associated with certain non-malignant diseases. As with conventional anti-CEA, the MA/1 antibodies can recognize significant amounts of CEA in serum from patients with a variety of solid tumours. However they appear to recognize a different immunodeterminant and possibly a different population of CEA molecules to, or a subset of, those measured by two routine assays. Studies in which the MA/1 assay was directly compared with the results of the Charing Cross routine and Abbott EIA assays have indicated that different immunological forms of CEA may be expressed in the course of tumour progression but no prognostic value was evident in this study. Our results stress the need to resolve immunological specificities expressed by CEA-like molecules and evaluate their clinical importance.**

SOMATIC-CELL HYBRIDIZATION, as used by Köhler & Milstein (1975), has proved to be a powerful tool for the *in vitro* production of specific monoclonal antibodies. The method has proved entirely successful for the production of antibodies to cell-surface antigens (Trucco *et al.*, 1978) and viruses (Koprowski *et al.*, 1977). It may also have great potential in the field of secreted tumour markers and monoclonal antibodies against CEA (Accolla *et al.*, 1979; Rogers *et al.*, 1981), HCG (Stahli *et al.*, 1980) and α-foetoprotein (Tsung *et al.*, 1980) have been described.

Monoclonal anti-CEA antibodies are of particular interest since they promise to overcome many of the difficulties of conventional sera. The need for extensive absorption of antibodies to normal components and to cross-reactive antigens before use in clinical radioimmunoassay would be eliminated, and there is the possibility of defining CEA and associated molecular forms more precisely. Moreover monoclonal antibodies should provide reagents with which to test the concept that different immunological forms of CEA could be expressed by different diseases (Rogers, 1976).

The major limitations of CEA assays in their present forms are: (1) failure to detect and monitor subclinical cancer, (2) failure to discriminate between cancer and certain non-malignant diseases, and (3) the variation in numerical values for CEA obtained with different assay systems (Vrba *et al.*, 1975).

We have recently reported monoclonal antibodies against CEA isolated from liver metastases of colonic tumour, and have determined conditions under which radioimmunoassay of CEA in human sera could be carried out (Rogers *et al.*, 1980, 1981). This paper describes the use of a radioimmunoassay using a monoclonal antibody, MA/l, to measure CEA in human serum, and reports preliminary results on the assessment of sera from normal subjects and patients with a variety of malignant and non-malignant diseases, a comparative study on colo–rectal and gastric cancer in which MA/1 assay values are compared with two CEA assays using heterologous antisera, and serial CEA measurements on patients with colo–rectal cancer as determined by the monoclonal assay and the two conventional assays. A part of this work has already been reported by Rogers *et al.* (1980).

### MATERIALS AND METHODS

*Patients' serum samples.*—Serum samples from normal individuals were taken from hospital personnel and medical students in the age group 19–40 years. Samples from patients with non-malignant disease were obtained from the Department of Pathology, Charing Cross Hospital. Other samples were taken from our sample bank, and composed of sera from patients routinely assessed for CEA and known to have malignant disease. Longitudinal studies were made on patients attending follow-up clinics following the resection of a primary colonic or rectal cancer. Unless otherwise stated, patients reported as having no evidence of recurrence throughout the period of study remained tumour-free for at least 6 months after the study had been completed.

*Assays for CEA.*—The monoclonal MA/1 assay was adapted to make use of the filtering and computing capabilities of the Kemtek 3000 automated RIA machine, though the assays were set up manually using standard dispensing equipment and 3ml polystyrene tubes. Samples and standards were assayed in triplicate by using 100 $\mu$l of test serum or CEA standards in assay buffer (phosphate-buffered saline 40 mM; EDTA 8 mM; bovine serum albumin, 1 g/l; thiomersal 20 mM, pH 7). One hundred $\mu$l of assay buffer was added to each serum sample and 100 $\mu$l of normal pooled serum (NPS) added to the standards. Fifty $\mu$l of monoclonal anti-CEA diluted 1:1000 and 50 $\mu$l of $^{125}$I-labelled CEA were then added to each tube. At the same time zero-antigen tests were set up in which 100 $\mu$l of assay buffer was added in place of the CEA standard. Nonspecific binding was determined by setting up tubes in which 50 $\mu$l of $^{125}$I-CEA, 150 $\mu$l of assay buffer and 100 $\mu$l of NPS were added. All tubes were incubated for 16 h at 37°C, and then 50 $\mu$l of rabbit anti-mouse Ig diluted 1:40 was added. (The rabbit anti-mouse serum was obtained from Dako, Product Z109 Batch 010A.) After a further 4 h at room temperature, the contents of each tube were filtered on to fibre-glass discs carried on the film of the Kemtek 3000. After washing and drying each disc was counted for $^{125}$I. The counting data were then fed to the microprocessor of the Kemtek 3000 to perform preprogrammed calculations. The concentration of CEA in each sample was calculated in units/ml by interpolation from the standard line. The standard line was based on a log–logit plot from counting data on the zero-antigen tests, the n.s.b. determination and from 9 CEA standards obtained by making sequential 2-fold dilutions from a 1000u/ml CEA standard (R41) (see Rogers *et al.*, 1980). The clinical cut-off chosen was 15 u/ml so that $>15$ u/ml was regarded as abnormal.

The Charing Cross routine assay using a conventionally prepared primary antiserum, PK1G (goat) raised to CEA Con A fraction 2B (R42) and absorbed with normal human spleen and normal human serum, was a double-antibody system similar to that described above. Samples were assayed directly without prior extraction. Precipitation of the CEA–anti-CEA complexes was achieved by using a second antibody (BW 402 horse anti-goat + sheep). CEA isolated by perchloric acid extraction and purification on Sepharose 6B and Sephadex G-200 (Preparation M12) was used as standard. CEA values below 10 ng/ml are regarded as normal. The normal range was determined by measuring 300 samples from patients with non-malignant diseases.

The Abbott EIA system was a solid-phase diagnostic immunoassay based on the sandwich principle (Oehr *et al.*, 1980). CEA present in the serum sample was extracted by heating in buffer (pH 5) at 85°C for 10 min. After centrifuging, the supernatants were assayed by binding the CEA to beads coated with guinea-pig anti-CEA. After washing, the beads were incubated with goat anti-CEA conjuaged with horseradish peroxidase. Colour intensity produced from the reaction of the enzyme with its substrate was measured, and the CEA (ng/ml) determined from the standard curve.

*Radiolabelled CEA.*—CEA Con A Fraction 2B (R42) was labelled with $^{125}$I (IMS 30, Radiochemical Centre, Amersham) by a modification of the Chloramine T technique (Greenwood *et al.*, 1963). Free iodine was removed by gel filtration on Sephadex G-200, and each fraction corresponding to the protein peak assessed for binding to the PK1G antiserum. The two fractions giving the highest binding of total counts added were pooled and used in the radioimmunoassay. A dilution giving 80,000 counts in 50 μl was used for the Charing Cross routine assay and a 1:200 dilution of the pooled label proteins corresponding to about 120,000 counts in 50 μl used for the MA/1 assay. The specific activity of the label (179 μCi/μg) was determined from the "self-displacement" analysis by the interpolated dose of the binding of several dilutions of label from the standard curve.

*Reagents.*—CEA (M-12) was isolated from a pool of 6 liver metastases of colonic tumour by perchloric acid extraction and chromatography on Sepharose 6B and Sephadex G-200, according to the method of Coligan *et al.* (1972). CEA-2B (R42) was prepared from a different pool of 6 liver metastases, as described above, and further purified on a column of Con A–Sepharose as previously described (Rogers *et al.*, 1976). The CEA standard (R41) was isolated from 6 additional specimens, as described for preparation R42.

*Units used in the MA/1 assay.*—Because of the poor inhibition by standard CEA preparations, an arbitrary unit has been adopted for use in the monoclonal MA/1 assay (Rogers *et al.*, 1981). Thus 100 u of MA/1-binding CEA is defined as that amount producing 50% displacement of bound label. In the case of the CEA standard (R41) 8600 ng/ml of CEA, as measured on the Charing Cross PK1G assay, was required for 50% displacement, and the top standard used was 43,000 ng/ml.

### RESULTS

An upper normal cut-off of 15 u/ml for the MA/1 assay was tentatively chosen on the basis of the assay results obtained with 144 sera from normal individuals and 200 sera from patients with various non-malignant diseases. Values > 15 u/ml were regarded as pathological. Of the normal sera, only 1 was > 15 u/ml, and 27 were 5–15 u/ml (Table I). Preliminary assessment of the sera from patients with a variety of non-malignant disease shows 5% of 161 raised if chronic renal failure was excluded (Table I). 25% of samples in the non-malignant group were in the range 5–15 u/ml and 70% below 5 u/ml. Of the 5% > 15 u/ml 2 were diabetic patients (23·5 and 25 u/ml), 2 had left ventricular failure (19·1 and 17·5 u/ml), one had undiagnosed chest pain (25 u/ml) and 2 had deep-vein thrombosis (29·3 and 18·6 u/ml). In another group of 43 patients with selected non-malignant disease, including chronic pancreatitis and hepatic cirrhosis, there were 5 with raised values (Table I). 33% of 40 samples from patients with chronic renal failure on the other hand were raised on the MA/1 assay, with values ranging from 15 to 55 u/ml (mean 24·5) (Table I).

Earlier studies (Rogers *et al.*, 1981) on the competitive binding of MA/1 have shown that 8,600 ng/ml of unlabelled CEA, extracted with perchloric acid from tumour tissue, was required to produce 50% inhibition of bound label, whereas only 30 ng/ml of CEA similarly extracted from patients' sera was required to pro-

TABLE I.—*Frequency of raised levels of MA/1-binding CEA in the serum of normal subjects and patients with non-malignant disease*

| Group | No. | (u/ml) < 5 | 5–15 | 15–20 | > 20 | % Raised (> 15 u/ml) |
|---|---|---|---|---|---|---|
| Normal subjects | 144 | 116 | 27 | — | 1 | 0·7 |
| Non-malignant, excluding chronic renal failure | 161 | 110 | 42 | 6 | 3 | 5 |
| Chronic renal failure | 40 | 13 | 15 | 4 | 9 | 33 |
| Chronic pancreatitis | 14 | 8 | 4 | 1 | 1 | — |
| Cirrhosis | 8 | 3 | 3 | 2 | — | — |
| Gastric and duodenal ulcer | 10 | 8 | 1 | 1 | — | — |
| Hepatitis | 5 | 3 | 2 | — | — | — |
| Gastritis | 6 | 3 | 2 | — | — | — |

TABLE II.—*Frequency of raised levels of MA/1-binding CEA in the serum of cancer patients*

| Location of tumour | No. patients | MA/1-binding CEA (u/ml) < 5 | 5–15 | 15–30 | 30–50 | > 50 | % Raised (> 15 u/ml) |
|---|---|---|---|---|---|---|---|
| Rectum | 95 | 30 | 35 | 12 | 4 | 14 | 31 |
| Colon | 102 | 48 | 25 | 13 | 4 | 12 | 29 |
| Stomach | 72 | 24 | 6 | 25 | 8 | 8 | 57 |
| Breast | 92 | 43 | 31 | 13 | 2 | 3 | 19 |
| Prostate | 34 | 8 | 14 | 3 | 4 | 5 | 35 |
| Lung | 79 | 43 | 24 | 3 | 6 | 3 | 15 |
| Ovary | 57 | 37 | 11 | 5 | 3 | 1 | 16 |
| Teratoma | 21 | 9 | 7 | 1 | 3 | 1 | 24 |
| Choriocarcinoma | 27 | 11 | 13 | 1 | 1 | 1 | 11 |

duce the same displacement. In view of these results it was necessary to determine whether the radioimmunoassay developed with MA/l could measure MA/l-binding CEA directly in the serum of patients with cancer. To check this, and also to assess the type of cancers which could be potentially followed on the monoclonal MA/l assay, random samples from several groups of patients were studied. Each group included samples from patients with early and metastatic cancer, but no data on the clinical status of the patients and response to treatment were considered for this preliminary assessment. Like conventional CEA, MA/l-binding CEA was found to be associated with many forms of cancer, but mainly with colo-rectal, gastric and prostatic cancer (Table II). Of a further 71 samples from patients with other cancers, too varied to classify, 41% were raised, the main groups including carcinoma of pancreas, urothelial cancer, oesophageal cancer and cervical cancer. With the exception of gastric cancer, in which 57% of 72 samples were raised, the incidence of raised values for all other cancer groups was similar to that obtained by measuring the same samples on the Charing Cross routine assay.

FIG. 1.—Chart showing assay values of those serum specimens from a total of 270 samples from patients with colo-rectal cancer, raised on the monoclonal assay (MA/l) but < 10 ng/ml on the Abbott assay; and those raised on the Abbott assay (> 10 ng/ml) but < 15 u on the MA/l assay. 78 of the 270 specimens (29%) were also raised on both assays and many of these showed discordant values (data not shown).

Of 83 specimens from patients with colo-rectal cancer, measured on both the Charing Cross routine assay and the monoclonal assay, the incidences of raised values were 33% and 30% respectively, though the values for many of the specimens differed. In particular, of the 83 specimens, 8 were raised on the monoclonal assay but < 10 ng/ml on the routine assay, and 10 were raised (> 10 ng/ml) on the routine assay but < 15 u/ml on the monoclonal assay. 20% of the specimens were raised on both assays, and a total of 42% were abnormal if both assays were combined. Furthermore, of 31 additional samples from patients with gastric cancer, 22% were raised on the Charing Cross routine assay, whereas 51% were raised on the monoclonal assay.

FIGS 2–8.—Serial studies in patients with colo-rectal cancer. △ = MA/1 assay; ● = Charing Cross routine assay; ○ = Abbott assay. Abbot and Charing Cross routine assay values are expressed in ng/ml whereas the MA/1 assay values are expressed in u/ml (see Materials and Methods). Broken line represents upper limit normality.

FIG. 2.—Patients in Group A.

Comparative data on 270 additional samples from patients with colo-rectal cancer, measured on the commercial Abbott diagnostic kit and monoclonal assay, have also shown that, although the incidences of raised values were similar for each assay (47% and 41% respectively) some of the pairs of results differed markedly. Of the 270 samples, 33 were raised on the monoclonal assay but < 10 ng/ml on the Abbott assay, and 42 were raised on the Abbott assay but < 15 u/ml on the monoclonal assay (Fig. 1).

*Serial studies*

Thirty-three patients with colo-rectal cancer were followed serially on the monoclonal, Charing Cross routine and Abbott assays for circulating CEA. Four main groups emerged from this study:

*Group A*.—Six patients have progressed favourably for at least 2 years after surgical resection of their primary tumours, and have shown no clinical evidence of recurrence during the assessment period. In 5 of these cases all 3 assay parameters have remained below the clinical cut-off limit during the follow-up .In the other case (Fig. 2A) the monoclonal and Charing Cross routine assays were transiently raised 3 months and 7 months after tumour resection, the latter rise correlating with adjuvant chemotherapy. Subsequent assay values on this patient have remained below the clinical cut-off, consistent with good clinical progress. One patient in this group had a significantly raised and rising $\gamma$-glutamyltranspeptidase (GGT) level, indicating impaired liver function, despite normal levels of circulating CEA and lack of evidence of tumour recurrence (Fig. 2B).

*Group B*.—Raised serum CEA levels were encountered in 3 patients without

FIG. 3.—Patients in Group B.

any evidence of clinical recurrence during the assessment period. In one case (Fig. 3) the patient had a mucus-secreting adenocarcinoma of the pelvic colon with omental secondaries which responded to treatment, and the patient was clinically clear 16 months later. During this period all 3 assays showed transient rises. This patient died 2 months after the end of the study, and had clinical evidence of recurrence in the last month before his death. In the other 2 patients the Abbott assay values

FIG. 4.

FIG. 5.

FIG. 6.

FIGS 4, 5, 6.—Patients in Group C.

were normal, whereas the Charing Cross and monoclonal assays were consistently raised over a period of several months. The significance of these responses is not known. The 3 patients in this group had normal $\gamma$-glutamyltranspeptidase levels.

Twenty-four patients in this study developed either recurrent or metastatic tumours or both during the assessment period, and these are considered in Groups C and D.

*Group C.*—In 8 of these patients there was a broad correlation between the monoclonal assay data and the results of the two conventional assays (Figs 4 and 5). In all cases progression of the disease and ensuing metastases was accompanied by a rising trend in the CEA values, though the rise was not always steady. Lack of samples precluded a detailed comparison of the onset of a positive value for each assay, but in 4 cases the monoclonal assay was raised somewhat later than the other two assays. One of these patients (Fig. 6) is interesting, since despite the Charing Cross routine and Abbott values being raised during a period where recurrent cancer was well controlled by chemotherapy, the monoclonal assay did not respond until the patient developed an intractable local recurrence in the pelvis and the disease started to progress. $\gamma$-Glutamyltranspeptidase levels in all but

FIG. 7.

FIG. 8.

FIGS 7, 8.—Patients in Group D.

one of the patients in this group were raised, correlating with metastatic spread into the liver.

*Group D.*—In 16 patients in this study, progression of the tumour was accompanied by only a partial assay response. In 7 of these cases the Abbott assay was not markedly raised at any stage of the disease, though marked transiently raised values were observed with either the Charing Cross assay or the MA/l assay or both. For one of these patients, the MA/l assay correlated with a symptomatic pelvic recurrence of tumour, whereas both the conventional assays were not raised at all. $\gamma$-Glutamyltranspeptidase levels in this patient were only moderately high and there was no clinical evidence of liver metastases. A marked transient rise and fall in the MA/l assay accompanied by imminent death of the patient (Fig. 7) was a noteworthy feature of this group. In some cases this type of pattern was accompanied by a similar pattern in the Charing Cross assay (Fig. 7), or by a rising CEA as measured in both conventional assays. In 2 cases all 3 assays values have remained below the cut-off limit for at least 18 months before death of the patient and during a period of progressive tumour invasion. In one of these cases the patient showed no clinical signs of recurrence, despite disseminated carcinomatosis proven *post mortem*. The $\gamma$-glutamyltranspeptidase levels in these patients were low, probably reflecting minimal liver involvement.

In the 9 cases in which the Abbott assay was markedly raised, the general pattern was a fairly steady increase in value with progressing disease (Fig. 8). In 6 of these cases a similar pattern was seen with the Charing Cross assay (Fig. 8). However, rising values on the conventional assays in these patients were frequently accompanied by either no response or a transient change in the MA/l assay. In these cases failure of the MA/l assay to reflect progressive tumour growth was usually accompanied by a normal or only moderately raised $\gamma$-glutamyltranspeptidase level, suggesting minimal liver involvement, and the possibility that much of the circulating CEA was originating from sites other than the liver.

### DISCUSSION

As with the conventional CEA assays, an arbitrary cut-off has been set on the monoclonal assay because of low levels of MA/l-binding CEA in serum from normal individuals. Further assessment of normal sera from a wider population and an extended age group may be warranted, but exclusion of occult disease in elderly subjects is difficult. With an upper normal limit of 15 u/ml the overall false-positive rate of the MA/l assay for non-malignant conditions was similar to that of the Charing Cross routine assay, in which non-malignant liver and colonic diseases were the most common conditions producing a raised value. However, the detailed comparison between the MA/l and the Charing Cross assays to measure non-malignant disease revealed marked numerical variations in the actual values which are probably attributable to the differing specificities of the two antisera. The association between raised CEA and chronic renal failure is noteworthy. Raised conventional CEA values associated with this condition have been previously reported in 37% of 27 patients with chronic renal failure who lacked evidence of either malignancy or other recognized non-malignant cause of the elevation (Brandstetter *et al.*, 1979). The reason for this is obscure, but in view of the unlikelihood of CEA or its immunologically active degradation products normally being cleared by the kidney, it could be attributed to an increased synthesis of CEA or diminished catabolism of the glycoprotein in these patients. The response of the MA/l assay and our routine assay to chronic renal failure differ markedly, however. Whereas the MA/l assay picks up 33% of 34 cases, the Charing Cross assay responds in only 3%. This is an interesting observation for, although we cannot completely exclude malignancy from the patients in this group, it could reflect a radical difference in specificity of these assays for at least one non-malignant condition.

The finding of substantial amounts of MA/l-binding CEA in the serum of patients with cancer has substantiated earlier evidence (Rogers *et al.*, 1981) that this monoclonal antibody detects a species of CEA more prevalent in serum than in tumour extracts. Other studies (Vrba *et al.*, 1975) have indicated that serum CEA may differ chemically and immunologically from CEA extracted from tumour tissue. The use of conventional polyvalent antisera may have previously obscured this difference.

The MA/l assay did not differ markedly from conventional CEA assays in its specificity for cancers according to site of origin. On the contrary, measurements on samples from many groups have shown a similar incidence of high values to that encountered in the Charing Cross assay, with the possible exception of gastric cancer, thus warranting further assessment of this group. The marked differences in response of individual assays described here for colo–rectal cancer, however, support the concept of differing immunological forms of CEA being expressed by different patients. The different spectra of positivity also suggest that an improved detection rate may be possible by using assays with several monoclonal antibodies selected for their specific characteristics.

It is worth noting at this point that the incidence of raised values (quoted in Table II) for any given cancer group will not necessarily agree with the statistics of other laboratories, as this depends on the particular samples assayed. The incidence of positivity of preoperative CEA levels is to some extent, depending on the specificity of the assay, related to post-operative staging of a tumour (Paone *et al.*, 1980). However, as our serial studies on patients with colo–rectal cancer have shown, the transitory nature of many values would make it difficult in this study to correlate progressing disease and incidence of positivity in a meaningful way.

Serial studies on 33 patients with colo–rectal cancer have permitted further observations. The prognostic reliability of a normal CEA level is not improved by the MA/l assay. It failed to produce a raised value at any stage of the disease in 7 patients with progressive cancer. By comparison, the Abbott assay was marginally raised (5–10 ng/ml) in 8 cases of progressive disease if a cut-off of 5 ng/ml was chosen, but at these low values no trend was seen. The Charing Cross assay value was consistently normal in only 3 of the cases of progressive disease in Group D. Where the value was raised it tended to be a transient change and only 4/16 cases in this group displayed a steadily rising trend. It may be concluded that the colo–rectal tumours in this group express different forms of CEA which are recognized differently by each of the 3 assays. Moreover, the results on individual patients show that different forms of CEA may be expressed at different stages of the disease and, unfortunately, this expression may not correspond to tumour progression.

A correlation between the circulating CEA level and progression of tumour spread, as determined clinically, was evident in the 8 cases of Group C, and here all 3 assay trends appeared broadly to correspond, though the MA/l assay responded somewhat later than the conventional assays in at least 4 cases. In contrast, in Group D a rising trend in at least one assay was found in only 8/16 cases. In most other cases there were transient changes in the assay response. These changes may reflect physiological factors influencing the growth rate of the tumour or biochemical manifestations influencing the production of CEA, its immunological expression or its appearance in the circulation. The clinical significance, if any, of these transient changes is obscure, though it has been noted that trauma following, for instance, peritoneal resection, can sometimes produce raised circulating CEA levels in the weeks that follow. The effects of chemotherapy could be another factor influencing the transport or detection of CEA in the circulation.

### CONCLUSIONS

Despite the relative inability of the monoclonal antibody MA/l to bind to CEA extracted from tumour tissue, this study has demonstrated the presence of substantial amounts of MA/l-binding CEA in the serum of some patients with cancer. Low levels are found in sera from normal individuals and moderately high levels are sometimes associated with certain non-malignant diseases. As with the conventional CEA assays, significantly raised values are found in serum from patients with a variety of solid tumours. Direct comparisons between the monoclonal and conventional assays have revealed marked differences in the actual assay values, indicating differences in specificity between the assays. The MA/l assay appears to measure a different population of CEA molecules or a subset of those measured on both routine assays. The results of serial measurements have further suggested that patients may express different immunological forms of CEA in the course of tumour progression, but no prognostic value was evident in this preliminary study. The results of this study stress the need to resolve the immunological specificities expressed by CEA-like molecules and evaluate their clinical importance. The development of monoclonal anti-CEA antibodies should facilitate this task considerably.

We are grateful to Professor G. R. Giles of St James's University Hospital, Leeds, for his permission to investigate patients under his care and to Mr R. Turner, for his technical assistance.

This work was supported by the Medical Research Council.

### REFERENCES

Accolla, R. S., Carnel, S., Phan, M., Henmann, D. & Mach, J. P. (1979) First report of the production of somatic cell hybrids secreting monoclonal antibodies specific for carcinoembryonic antigen (CEA). *Protides Biol. Fluids*, **27**, 31.

Brandstetter, R. D., Graziane, V. A., Wade, M. J. & Saal, S. D. (1979) Carcinoembryonic antigen elevation in renal failure. *Ann. Intern. Med.*, **91**, 867.

Coligan, J. E., Lautenschleger, J. T., Egan, M. L. & Todd, C. W. (1972) Isolation and characterisation of carcinoembryonic antigen. *Immunochemistry*, **9**, 377.

Greenwood, F. C., Hunter, W. M. & Glover, J. S. (1963) The preparation of $^{131}$I-labelled growth hormone of high specific activity. *Biochem. J.*, **89**, 114.

Köhler, G. & Milstein, C. (1975) Continuous cultures of fused cells secreting antibody of predefined specificity. *Nature*, **256**, 495.

Koprowski, M., Gerhard, W. & Croce, C. M. (1977) Production of antibodies against influenza virus by somatic cell hybrids between mouse myeloma and primed spleen cells. *Proc. Natl Acad. Sci., U.S.A.*, **74**, 2985.

Oehr, P., Schlösser, T. & Adolphs, H. D. (1980) Applicability of an enzymic test for the determination of CEA in serum and CEA-like products in urine of patients with bladder cancer. *Tumor Diagnostik*, **1**, 40.

Rogers, G. T. (1976) Heterogeneity of carcinoembryonic antigens: Implications on its role as a tumour marker substance. *Biochim. Biophys. Acta*, **458**, 355.

Rogers, G. T., Searle, F. & Bagshawe, K. D. (1976) Carcinoembryonic antigen: Isolation of a sub-fraction with high specific activity. *Br. J. Cancer.*, **33**, 357.

Rogers, G. T., Rawlins, G. A. & Bagshawe, K. D. (1980) Monoclonal antibodies against carcinoembryonic antigen (CEA). *Protides Biol. Fluids*, **28**, 517.

PAONE, J. F., KARDANA, A., ROGERS, G. T., DHAZMANA, J. & JEYASINGHAM, K. (1980) Preoperative carcinoembryonic antigen levels correlated with postoperative pathological staging in bronchial carcinoma. *Thorax*, **35**, 920.

ROGERS, G. T., RAWLINS, G. A. & BAGSHAWE, K. D. (1981) Somatic cell hybrids producing antibodies against CEA. *Br. J. Cancer*, **43**, 1.

STAHLI, C., STACHELIN, T., MIGGIANO, V., SCHMIDT, J. & HARING, P. (1980) High frequencies of antigen-specific hybridomas. Dependence on immunisation parameters and prediction by spleen cell analysis. *J. Immunol. Meth.*, **32**, 297.

TRUCCO, M. M., STOCKER, J. W. & CAPPELLINI, R. (1978) Monoclonal antibodies against human lymphocyte antigens. *Nature*, **273**, 666.

TSUNG, Y.-K., MILUNSKY, A. & ALPERT, E. (1980) Secretion by a hybridoma of antibodies against human α-fetoprotein. *N. Engl. J. Med.*, **302**, 180.

VRBA, R., ALPERT, E. & ISSELBACHER, K. J. (1975) Carcinoembryonic antigen: Evidence for multiple antigenic determinants and isoantigens. *Proc. Natl Acad. Sci., U.S.A.*, **72**, 4602.